# Interferon gamma in cancer immunotherapy

**DOI:** 10.1002/cam4.1700

**Published:** 2018-07-23

**Authors:** Ling Ni, Jian Lu

**Affiliations:** ^1^ Institute for Immunology and School of Medicine Tsinghua University Beijing China; ^2^ Department of Urology Peking University Third Hospital Beijing China

**Keywords:** cancer immunotherapy, IFNγ signaling, immune evasion, immune surveillance, transformed self

## Abstract

Immune system can recognize self vs transformed self. That is why cancer immunotherapy achieves notable benefits in a wide variety of cancers. Recently, several papers reported that immune checkpoint blockade therapy led to upregulation of IFNγ and in turn clearance of tumor cells. In this review, we conducted an extensive literature search of recent 5‐year studies about the roles of IFNγ signaling in both tumor immune surveillance and immune evasion. In addition to well‐known functions, IFNγ signaling also induces tumor ischemia and homeostasis program, resulting in tumor clearance and tumor escape, respectively. The yin and the yang of IFNγ signaling are summarized. Thus, this review helps us to comprehensively understand the roles of IFNγ in tumor immunity, which contributes to better design and management of clinical immunotherapy approaches.

## 1 INTRODUCTION

Cancer is characterized by the accumulation of a growing body of genetic alternations and the loss of normal cellular modulation.^1^ The immune system can recognize not limited solely to the classic models of self vs pathogen or self vs nonself, but also self and transformed self.^2^ That is why cancer immunotherapy has demonstrated efficacy and achieved notable benefits in a variety of cancers. Several publications have demonstrated that CTLA‐4 and PD‐1 inhibitors as well as other immune checkpoint blockade therapies result in an increase in IFNγ production,^3^, ^4^, ^5^ which in turn lead to the elimination of cancer cells. Recently, Mauguso et al^6^ further confirmed that resistance to immunotherapy is attributed to defects in IFNγ signaling. These indicate that cancer immunotherapy acts at least partially through an increase of IFNγ expression.

IFNγ was first identified based on its in vitro antiviral activity. Its receptor consists of two subunits, IFNGR1 and IFNGR2.^2^ The ligation to its receptor leads to the recruitment and activation of the Janus kinase, JAK1 and JAK2, which resultantly activates STAT1 and interferon regulatory factor (IRF) 1. Phosphorylated STAT1 and IRF1 translocate to the nucleus, where they bind to specific promoter elements and modulate transcription of IFNγ‐regulated genes. Recently, IFNγ has been shown to have obligate roles in cancer immunology.^2^ In this review, we have conducted an extensive literature search of recent 5‐year studies of the roles of IFNγ signaling in the immune responses in cancer patients as well as in the tumor‐bearing mice. The objective is to assess the contributions of IFNγ signaling to host protection. In the meantime, the role of IFNγ signaling in tumor escape from immune elimination is discussed. The yin and the yang of IFNγ signaling are summarized. Furthermore, IFNγ as an anticancer drug and clinical trials involving IFNγ alone or in combination with other anticancer drugs are also discussed. Thus, this review helps us to comprehensively understand the roles of IFNγ in antitumor immune response and protumor escape, which contributes to better design and management of clinical immunotherapy approaches.

## 2 IFNγ EXPRESSION AND SIGNIFICANCE IN CANCER

IFNγ is produced predominantly by T cells and NK cells in response to a variety of inflammatory or immune stimuli. For example, inflammasome activation leads to the maturation and secretion of IL‐18. The ligation of IL‐18 to its receptor activates MyD88 signaling pathway, which resultantly induces IFNγ production.^7^ In the context of tumor, tumor‐infiltrating lymphocytes (TILs) are the main source of IFNγ, which have shown of particular importance in tumor immunosurveillance. Recently, there are several papers regarding factors that can regulate IFNγ expression in tumor‐infiltrating NK cells and T cells. One factor is lactate acidosis, a hallmark of malignant tissue, which negatively regulates IFNγ production by NK cells in the context of tumor transformation.^8^ B‐cell lymphoma development was accompanied by decreased pH values and lactate accumulation in the growing tumor microenvironment, which could result in progressive loss of IFNγ expression in NK cells.^8^ Moreover, transfer of lymphoma‐derived NK cells into a normal micromilieu could rescue IFNγ production in these transferred cells.^8^ Likewise, treatment of lymphoma‐bearing mice with systemic alkalization by oral delivery of bicarbonate leads to enhancing IFNγ production by NK cells and increasing numbers of NK cells in the lymphoid organs.^8^ These suggest that reduced pH values and lactate accumulation in tumor microenvironment can downregulate IFNγ expression by NK cells.

Another factor is epigenetic modification. Wang et al^9^ showed that plasma IFNγ levels were significantly decreased in lung cancer patients and hypermethylation of the IFNγ promoter in CD4^+^ T cells was inversely associated with plasma IFNγ levels. Moreover, CD4^+^ T cells from healthy donors cocultured with SPC‐A1 cells (lung cancer cell line) resulted in a reduction in IFNγ expression after stimulation, an increase in DNA methyltransferases (DNMTs) and hypermethylation of the IFNγ promoter. Thus, a reduction in IFNγ expression of CD4^+^ T cells cocultured with lung cancer cell is correlated with the hypermethylation of IFNγ promoter.^9^ These findings suggest that interaction between lung cancer cells and CD4^+^ T cells induces DNMT expression and hypermethylation of IFNγ promoter in CD4^+^ T cells, which silence IFNγ gene expression.

MicroRNA‐155 (miR‐155) is another factor that upregulates IFNγ expression in the tumor microenvironments and slows tumor growth.^10^, ^11^ Huffaker et al reported a defect in the accumulation of IFNγ‐expressing CD4^+^ and CD8^+^ T cells in the tumors from miR‐155 knockout mice, indicating miR‐155 has tumor regression activity. In addition, miR‐155 can target and repress IFNγ regulator Ship1 to increase IFNγ expression by CD4^+^ T cells.^10^ These findings indicate that an increase in miR‐155 expression can be exploited to improve cancer immunotherapy. Twist1 negatively regulates IFNγ expression.^12^ Mechanistically, Twist1 can form a complex with runt‐related transcription factor 3 (Runx3) to reduce the binding of Runx3 and T‐bet to Ifng locus, which resultantly suppresses IFNγ expression.^12^ In a mouse model of sporadic colon cancer, IL‐33 treatment induces IFNγ secretion by tumor allograft‐infiltrating T cells and the deficiency of its receptor ST2 within the nonhematopoietic cells resulted in a reduced IFNγ gene expression signature.^13^ However, the mechanism underlying IL‐33‐induced IFNγ expression is needed to investigate.

The clinical significance of IFNγ expression in human cancer has been observed. Higgs et al^14^ found that in patients with metastasized NSCLC and urothelial cancer who have been received PD‐L1 inhibitor (durvalumab), an increased IFNγ gene signature (*IFNγ, CD274, LAG3,* and *CXCL9*) is correlated with higher overall response rates and longer median progression‐free survival, which is independent of PD‐L1 expression assessed by immunohistochemistry, suggesting that IFNγ gene signature may stratify patients with improved outcomes to anti‐PD‐L1 antibodies. Furthermore, one recent report showed that PD‐1 inhibitor treatment of NSCLC patients and melanoma patients leads to higher IFNγ protein expression, accompanying with significantly longer progression‐free survival,^15^ indicating that IFNγ could be a biomarker for prediction of response to immune checkpoint blockade. However, in patients with locally advanced lung adenocarcinoma, tumor‐expressing IFNγ alone has no significant prognostic value, while tumor‐expressing both IFNγ and PD‐L1 have the best value.^16^ This discrepancy could be due to cancer patient heterogeneity, tumor stage or tumor type.

## 3 THE ROLE OF IFNγ IN IMMUNE ELIMINATION

Ionizing radiation, one of traditional cancer treatments, works primarily through the induction of tumor cell damage at a molecular level. Recently some studies have shown that the immune system is required for effective radiotherapy and IFNγ plays a pivotal role in the efficacy of ionizing radiation therapy.^17^, ^18^ In a mouse colon cancer model, ionizing radiation therapy has no effect on tumor growth in IFNγ KO mice, but decreases tumor burden in WT mice. This could be because ionizing radiation treatment enhanced the capacity of T cells to lyse tumor cells, which is dependent on IFNγ.^17^ This finding suggests that IFNγ gets involved in mediating the antitumor effects of ionizing radiation therapy.

In addition, IFNγ decreases tumor cell growth by inducing tumor cell cycle arrest, apoptosis and necroptosis. In cancer types, such as breast cancer,^19^ colorectal cancer^20^ and hepatocellular cancer,^21^ IFNγ can exert antiproliferative effect on tumor cells by enhancing expression of the cell cycle inhibitor proteins p27Kip, p16 or p21. Moreover, in colorectal cell lines, IFNγ elicits autophagy‐associated apoptosis through induction of mitochondria‐derived reactive oxygen species (ROS), which is dependent on cytosolic phospholipase A2 (cPLA2) activation.^22^ However, in melanoma cell lines, IFNγ induces a increase in miR‐29a/b, which is STAT1 dependent, but not cell cycle inhibitor proteins, and there is a negative correlation between miR‐29a/b expression and the proliferation rate of various cell lines.^23^ Furthermore, G1‐arrest of melanoma cells induced by IFNγ requires decreased expression of cyclin‐dependent kinase 6 (CDK6), which is a direct target of miR‐29 in these cells.^23^ In certain samples from patients with primary melanoma, but not metastatic melanoma or normal skin, the expression levels of miR‐29a and miR‐29b are found dramatically increased,^23^ suggesting that IFNγ decreased melanoma cell growth by arresting tumor cell cycle via miR‐29a/b upregulation. Recently, Cekay et al^24^ showed that a novel synergistic interaction of IFNγ with second mitochondria‐derived activator of caspases (Smac) mimetics that antagonize x‐linked Inhibitor of Apoptosis (XIAP) to induce necroptosis in apoptosis‐resistant cancer cells where caspase activation is suppressed. This synergistic effect is observed in both solid and hematological cancer cell lines as well as for different Smac mimetics, indicating a broader relevance.^24^

IFNγ also has to act on the tumor stroma for effective elimination of large, established tumors, although it can inhibit tumor growth by acting directly on cancer cells. Recently Kammertoens et al^25^ showed that responsiveness of myeloid cells and other haematopoietic cells to IFNγ was not sufficient for tumor regression induced by IFNγ, whereas responsiveness of endothelial cells to IFNγ was necessary and sufficient for tumor regression. On the mechanism, IFNγ elicits regression of the tumor vasculature, leading to arrest of blood flow and subsequent collapse of tumors, which is like non‐hemorrhagic necrosis in ischemia and unlike hemorrhagic necrosis induced by TNFα.^25^ This finding suggests that IFNγ slow tumor growth via induction of tumor ischemia.

For immune cells, IFNγ signaling activates antigen‐presenting cells (APCs) to upregulate the expressions of cytokines (IL‐12 and IL‐18) and costimulatory molecule CD86 that enhance Th1 differentiation and cytotoxic T lymphocyte (CTL) function.^26^, ^27^, ^28^ Furthermore, those APCs activated by IFNγ increase the expression of MHC molecules and components of the antigen‐processing machinery. In addition, IFNγ induces a number of signals in T cells to enable T cell function effectively, while the loss of IFNγ signaling pathways in T cells dampens T cell responses and allows tumor growth and persistence.^29^ On the other hand, IFNγ signaling also promotes tumor elimination by inhibiting the functions of some suppressive immune cells in the tumors, such as regulatory CD4^+^ T cells (Tregs),^30^ myeloid‐derived suppressor cells (MDSCs)^31^ and tumor‐associated macrophages (TAMs).

Tregs permit tumor growth and are a barrier in an effective antitumor immune response. Neuropilin‐1 (Nrp1), a trans‐membrane molecule, is needed to maintain the stability and function of tumor‐infiltrating Tregs but is dispensable for peripheral Tregs.^30^ Overacre‐Delgoffe et al^30^ recently found that a high frequency of Nrp1^−/−^ Tregs in the tumors produce IFNγ, which suppress surrounding WT Tregs in the tumor and in turn facilitated tumor elimination. In addition, intratumoral Treg fragility induced by IFNγ contributes to response to PD‐1 inhibitors, suggesting that IFNγ‐induced Treg fragility was required for an effective response to PD‐1‐targeting immunotherapy.^30^

Myeloid‐derived suppressor cells are present in most of the cancer patients. They also a major obstacle to antitumor immunity due to their capacity of inducing antigen‐specific CD8^+^ T‐cell tolerance through tyrosine nitration of TCR/CD8 complex.^32^ However, Medina‐Echeverz et al showed that IFNγ secreted by antigen‐specific CD8^+^ T cells induces a decrease in Bcl2a1 expression through a direct interaction of pSTAT1 with the Bcl2a1 promoter. Moreover, upregulation of Bcl2a1 in MDSCs results in prolonged survival and enhanced their suppressive function. Thus, IFNγ/STAT1 negatively regulates survival and thereby suppressive function of MDSCs via Bcl2a1,^31^ which may promote antitumor immune responses. However, the role of IFNγ in immune modulation of tumor microenvironment by MDSCs remains unexplored.

M1 macrophages enhance tumor regression, whereas M2 macrophages improve tumor progression. Monocytes‐derived TAMs are the M2‐polarized macrophages in most human tumors, which secrete a large amount of vascular endothelial growth factor (VEGF) to promote tumor growth.^33^ IFNγ can suppress the differentiation of monocyte‐derived TAMs in the tumor microenvironments and furthermore switch TAMs from M2 into M1 macrophages, which suppress VEGF secretion in vitro and in vivo and thereby inhibit angiogenesis. IFNγ exposure also switched THP‐1‐derived macrophages to the M1‐like macrophages with enhancing pro‐inflammatory capacity.^34^ In a mouse model of ovarian cancer, IFNγ and GM‐CSF by T cells activated TAMs to increase IL‐12p40 production and augment antigen processing and presentation to tumor antigen‐specific T cells.^35^ Moreover, IFNγ produced by T cells, but not GM‐CSF, induces macrophages to produce nitric oxide (NO) and enhances macrophage lysis of tumor cells.^35^ Collectively, these data suggest that IFNγ can re‐educate TAMs and switch them to M1 macrophages, which in turn promotes tumor elimination. On the other hand, in mouse melanoma model, the Src homology 2 domain‐containing protein tyrosine phosphatase 2 (Shp2) in macrophages promotes tumor development.^36^ Shp2 deficiency in macrophage induces CXCL9 following exposure to IFNγ treatment. CXCL9 further promotes tumor infiltration of IFNγ‐expressing T cells that resultantly enhance CXCL9 expression within tumor microenvironments.^36^ Thus, targeting Shp2 in macrophages may create Th1‐dominant tumor microenvironments.

**Figure 1:**
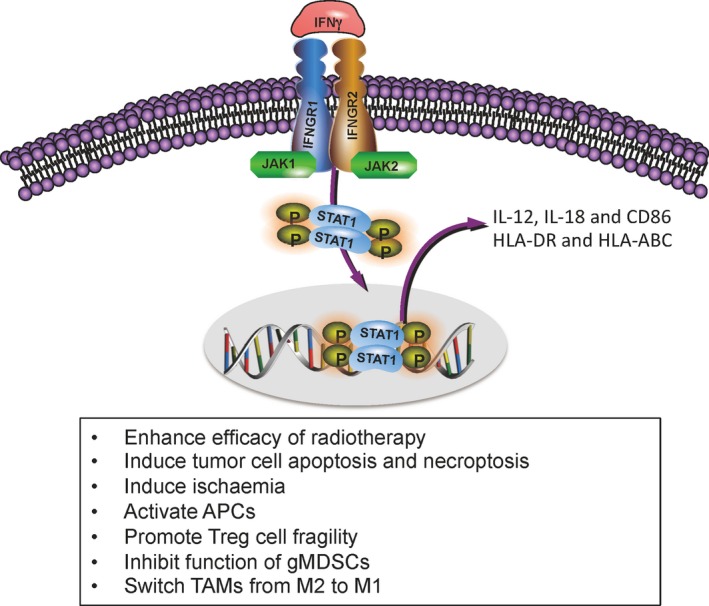
The roles of IFNγ signaling in tumor clearance. IFNγ signaling activates STAT1. Phosphorylated STAT1 binds to specific promoter elements and modulate transcription of IFNγ‐regulated genes. The positive consequences of IFNγ ligation consist of increased efficacy of radiotherapy, induction of tumor cell apoptosis and necroptosis, generation of ischemia, activation of APCs, promotion of Treg cell fragility, inhibition of gMDSC function and switch M2 from TAMs

Taken together, IFNγ can decrease tumor growth by acting not only directly on cancer cells, but also indirectly on endothelial cells and immune cells in the tumor microenvironments (Figure 1).

## 4 THE ROLE OF IFNγ IN TUMOR ESCAPE

**Figure 2:**
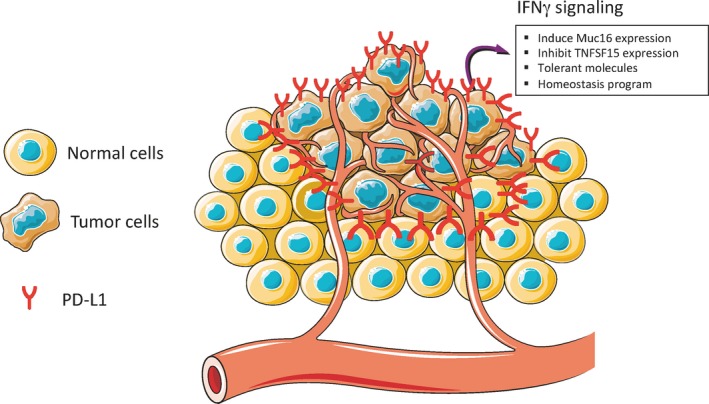
The roles of IFNγ signaling in tumor escape. IFNγ signaling induces tumor cells to express tolerant molecules, such as PD‐L1, which functions as a molecular shield to protect PD‐L1^+^ tumor cells from immune attack, while downregulates TNFSF15 to promote angiogenesis. In addition, IFNγ signaling induces Muc16 expression and homeostasis program to promote tumor progression

The above findings provide strong evidences that IFNγ plays a pivotal role in host antitumor immunity. However, IFNγ also contributes to the subsequent cancer evasion by promoting tumorigenesis and angiogenesis, eliciting expression of tolerant molecules and inducing homeostasis program (Figure 2).

MUC16 (also known as CA125) is a high molecular weight trans‐membrane mucin, a well‐known biomarker for human cancers.^37^, ^38^ MUC16 contributes to tumor development through multiple different mechanisms, such as suppressing NK cell killing capacity, reducing the sensitivity of cancer cells to drug therapy, and promoting cancer cell motility and so on.^38^ Morgado et al^37^ showed that IFNγ plus TNFα resulted in upregulation of MUC16 mRNA and protein in a wide spectrum of cancer cell types, but not alone, implying that this may be a general response. Furthermore, MUC16 expression is directly correlated with TNFα and IFNγ staining intensity in certain cancers.^37^ These data suggest that IFNγ signaling plus TNFα signaling promote tumorigenesis via MUC16. In addition, IFNγ also induces epithelial‐mesenchymal transition (EMT) in human papillary thyroid cancer (PTC) cells and increases the migratory and invasive behavior of PTC cells.^39^ This indicates that IFNγ induces EMT and promotes adverse outcomes in PTC.

TNFSF15 maintains blood vessel stability through negatively modulating neovascularization.^40^ *TNFSF15* gene expression decreases at angiogenesis and inflamed sites such as cancers. Lu et al^41^ showed that IFNγ/STAT1 signaling pathways suppressed TNFSF15 expression in human umbilical vein endothelial cells and TNFSF15 expression diminished while tumor vascularity increased in ovarian cancer clinical specimens with high levels of IFNγ expression.^41^ This finding indicates that IFNγ produced by tumor microenvironments inhibits TNFSF15 expression in vascular endothelial cells, leading to angiogenesis in the tumors.

Induced nitric oxide synthases (iNOS) can produce NO and NO contributes to the suppressive activity of monocytic MDSCs (CD11b^+^Ly6G^−^Ly6C^+^) and macrophages. Shime et al showed that IFNγ produced by CD8^+^ T cells can elicit iNOS expression in monocytic MDSC‐derived macrophages, but not undifferentiated monocytic MDSCs. In addition, iNOS plays a pivotal role in enhancing suppressive activity of TLR2 ligand‐treated monocytic MDSCs and in turn reducing antitumor T‐cell responses. These findings indicate that IFNγ induced iNOS and TLR2 ligand enhance the immunosuppressive capacity of monocytic MDSCs, which may downregulate antitumor CTL response.^42^

IFNγ induces the expression of some tolerant molecules, such as CTLA‐4, PD‐L1 and indolamine‐2,3‐ dioxygenase‐1 (IDO1) on/in tumor cells. CTLA‐4 is one of immune checkpoint molecules expressed on T cells and CTLA‐4 inhibitor is dramatically effective at restoring T‐cell responses in the patients with melanoma. Recently, Mo et al^43^ reported that IFNγ induced melanocyte and melanoma cells to express human CTLA‐4 gene, which was dependent on IFNGR/STAT1 signaling pathways. More interestingly, CTLA‐4 inhibitor (ipilimumab) therapy leads to an increase in an IFNG‐response gene expression signature in melanoma patients, including CTLA‐4 itself.^43^ However, there is an urgent need to investigate the function of CTLA‐4 on melanoma cells in tumor immune escape.

PD‐L1 expression is elicited by multiple cytokines, of which IFNγ is the most potent.^44^ Under the physical condition, IFNγ‐induced PD‐L1 expression on APCs and other cells maintain the threshold of T‐cell activation to avoid damage of tissue and organ. Under cancer condition, PD‐L1 expression is a strategy exploited by tumor cells to escape antitumor immunity. Established human tumor cell lines rarely express surface PD‐L1, but IFNγ treatment can induce most of the cell lines to express high levels of surface PD‐L1. In addition, normal epithelial cells, vascular endothelial cells and proximal tubular epithelial cells can also be induced to express high levels of PD‐L1 by IFNγ. Bellucci et al^45^ showed that increased expression of PD‐L1 by tumor cells resulted in enhanced resistance to NK cell lysis, while blockade of IFNγ/JAK signaling pathway leads to higher tumor cell lysis mediated by NK cells. In addition, IFNγ treatment of gastric tumor cell lines followed by PD‐L1 antibody results in enhancing antitumor CTL activity.^46^ Moreover, in clinical gastric cancer samples, PD‐L1 expression on tumor cells is significantly associated with IFNγ expression in the tumor and the proportion of CD8^+^ T cells in the stroma.^46^ These findings imply that gastric cancer patients with high CD8^+^ T‐cell infiltration and intratumoral IFNγ expression may be more responsive to PD‐L1 inhibitor therapy.

However, Gao et al^16^ observed that treatment of lung adenocarcinoma cells with IFNγ led to activation of JAK2‐STAT1 and PI3K‐AKT pathways. JAK2‐STAT1 activation contributes to IFNγ antiproliferative effect while PI3K‐ACT activation induces PD‐L1 expression and decreases IFNγ antiproliferative effect, implying that blockade of PI3K might maximize the IFNγ mediated antitumor effect.^16^ These findings indicate a crosstalk between JAK2‐STAT1 and PI3K‐AKT pathways in response to IFNγ in lung adenocarcinoma. However, these data are inconsistent with other previous observations that IFNγ induce PD‐L1 expression on tumor cells via activation of JAK/STAT signaling pathway. This discrepancy of PD‐L1 expression through signaling pathways should be further investigated.

Another tolerant molecule is IDO1, a kynurenine pathway enzyme, which is expressed by tumor cells to evade a potential effective immune response. High levels of IDO1 expression are correlated with poor prognosis in a wide spectrum of cancer types. IFNγ induces high levels of IDO1 in both human renal cell carcinoma and murine renal cell adenocarcinoma.^47^ It is known that IFNγ signaling elicits apoptosis of differentiated tumor cells via STAT1. However, Liu and colleagues showed that IFNγ signaling resulted in IDO1/AhR‐dependent p27 induction when IDO1 and AhR were highly expressed in tumor‐repopulating cells (TRCs).^48^ The p27 in turn bound to cytosolic pSTAT1, which prevented STAT1‐mediated tumor cell apoptosis. Blockade of the IDO/AhR metabolic circuitry not only abrogates dormancy induced by IFNγ, but also leads to increased tumor regression.^48^ These findings uncover a previously unrecognized mechanism underlying IFNγ‐induced TRC dormancy, implying a potential effective combination of IFNγ inhibitors with IDO/AhR inhibitors.

Immune protection and self‐tolerance are balanced by homeostatic program. Autoimmunity and tumor formation are likely impacted by such mechanisms, respectively. The recent finding 49 demonstrated that different immune mononuclear phagocytes shared a conserved steady‐state program during differentiation and entry into healthy tissue. More interestingly, IFNγ is sufficient to induce the conserved program. Furthermore, IFNγ‐induced and homeostatic programs enrich across primary human tumors and stratify survival. IFNγ could induce expression of suppressor‐of‐cytokine‐2 (SOCS2) protein, a conserved program transcript, which is expressed by mononuclear phagocytes infiltrating primary melanoma. SOCS2 limits adaptive antitumor immunity and DC‐based priming of T cells in vivo.^49^ These results link immune homeostasis to key determinants of antitumor immunity and escape, uncovering the underlying mechanism by which IFNγ contributes to tumor escape in the tumor microenvironments.

## 5 CONCLUSION

In the context of ovarian cancer, IFNγ in combination with cyclophosphamide and cisplatin significantly prolongs progression‐free survival.^50^ Moreover, there are 8 ongoing clinical trials involving IFNγ alone or in combination with other anticancer drugs up to now (Table 1).

**Table 1:** Ongoing clinical trials involving IFNγ alone or in combination with other anticancer drugs

NCT No.	Status	Conditions	Interventions	Locations	Phase
NCT02948426	Recruiting	Fallopian Tube Cancer, Ovarian Cancer, Primary Peritoneal Cancer	Autologous Monocytes + IFNγ + IFNα	National Institutes of Health Clinical Center Bethesda, Maryland, United States	Phase 1
NCT03112590	Recruiting	Breast Cancer	IFNγ with paclitaxel, trastuzumab and pertuzumab	H. Lee Moffitt Cancer Center and Research Institute Tampa, Florida, United States	Phase 1 Phase 2
NCT02614456	Recruiting	Advanced Solid Tumors	IFNγ and nivolumab	Fox Chase Cancer Center Philadelphia, Pennsylvania, United States	Phase 1
NCT02197169	Active, not recruiting	Glioblastoma or Gliosarcoma	IFNγ and DNX‐2401	Moffitt Cancer Center Tampa, Florida, United States The Ohio State University Columbus, Ohio, United States Baylor University: Charles A. Sammons Cancer Center Dallas, Texas, United States UT MD Anderson Cancer Center Houston, Texas, United States	Phase 1
NCT01957709	Recruiting	Myxoid Liposarcoma, Round Cell Liposarcoma, Synovial Sarcoma	Recombinant IFNγ	Fred Hutch/University of Washington Cancer Consortium Seattle, Washington, United States	A pilot study
NCT03063632	Recruiting	Recurrent Mycosis Fungoides and Sezary Syndrome, Refractory Mycosis Fungoides, Stage IB Mycosis Fungoides and Sezary Syndrome AJCC v7	IFNγ ‐1b and Pembrolizumab	University of Pennsylvania/Abramson Cancer Center Philadelphia, Pennsylvania, United States Cancer Immunotherapy Trials Network Seattle, Washington, United States	Phase 2
NCT03056599	Recruiting	Soft Tissue Sarcoma Adult	IFNγ with other anticancer drugs	Fred Hutchinson Cancer Research Center Seattle, Washington, United States University of Washington Seattle, Washington, United States	Phase 1
NCT02550678	Recruiting	Basal Cell Nevus Syndrome, Skin Neoplasm Nodular, Basal Cell Carcinoma of Skin	ASN‐002 (adenoviral particles carrying a gene coding for the human IFNγ) Alone or in Combination With 5‐FU	St George Dermatology and Skin Cancer Centre Kogarah, New South Wales, Australia Siller Medical T/A Central Brisbane Dermatology Brisbane, Queensland, Australia Veracity Clinical Research Brisbane, Queensland, Australia Sinclair Dermatology Melbourne, Victoria, Australia	Phase 1 Phase 2

**Figure 3:**
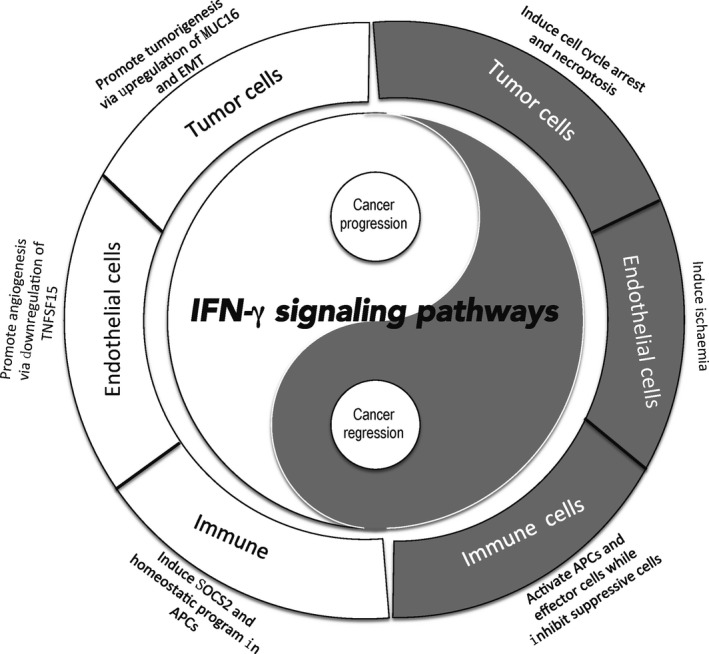
The yin and the yang of IFNγ signaling in cancer immunity. IFNγ plays a dual and opposing role in cancer development. IFNγ signaling inhibits tumor growth by arrest of tumor cell cycle, induction of tumor ischemia and activation of APCs and effector cells while impairing suppressive immune cells. Meantime, IFNγ contributes to tumor growth via promotion of tumorigenesis and angiogenesis, upregulation of tolerant molecules and induction of homeostasis program

Even though it's pivotal importance in cancer immunotherapy, IFNγ has not been approved by FDA to treat patients with a variety of cancer types, except malignant osteoporosis. This could be explained by the contribution of IFNγ to tumor evasion. The yin and the yang of IFNγ signaling in cancer immunity are summarized in Figure 3. A better understanding of the roles of IFNγ in tumor escape and tumor elimination will better design clinical immunotherapy approaches and provide new insights into cancer biology.

## CONFLICT OF INTEREST

The authors declare that there is no conflict of interest to disclose.
